# The Claims of Tuberculosis

**Published:** 1910-05-07

**Authors:** 


### The Claims of Tuberculosis.

The approved anti-tuberculosis plan of campaign already includes the following expensive measures: First, dispensaries worked in conjunction with a notification system, and equipped with a visiting staff of officials; secondly, sanatoria for patients who have a fair chance of regaining working capacity, supplemented with labour colonies for convalescents; thirdly, homes in which to isolate advanced cases, and possibly separate ones for the dying; fourthly, and more costly even than all the rest put together, pecuniary support of dependents upon the consumptive breadwinner. Many persons of experience, who would energetically repudiate any sympathy with socialistic schemes, demand this programme in its entirety in the most matter-of-fact way possible. Now, as it is obvious that for the next twenty years, at any rate, the only practicable way of financing the campaign as a whole will be by some big insurance provision, and also that the political situation gives no promise of any such scheme being determined upon for some considerable time, it must surely behove those responsible for the management of institutions for the benefit of consumptives to practise the strictest economy of their resources, with the object of doing as much as they can on the lines indicated above.

Limited enough these resources are, as a glance through Dr. Bulstrode's well-known report to the Local Government Board shows plainly, and the reason is not far to seek. Asylums and Poor Law institutions are State supported, while hospitals have a traditional claim upon private charity; but the testator and the philanthropist have not yet got sanatoria into their minds as possible objects of benefaction. Then, too, the length of time required for treatment is exceptionally great, and the diet required a generous one, much exceeding in cost that usually given to the sick.

In view of all this, it is strange to read some recent appeals by the authorities of certain sanatoria for comparatively large sums in order to build elaborate chapels and halls. Sacerdotalists may insist on the urgent necessity for supporting such appeals, but then sacerdotalists are out of place in the direction of medical concerns especially bound to benefit their patients to the maximum extent at the minimum cost. Already at one sanatorium a vast sum has been spent on such purposes, swelling the building cost per bed, as we pointed out not long ago, to a figure which is absolutely gigantic. To anyone acquainted with sanatorium work it is absolutely certain that there exist plenty of legitimate calls for expenditure without more going on bricks and mortar, materials which seem, moreover, not to be in demand in the latest types of sanatorium construction. No; true virtue lies, to quote the old Aristotelian dictum, in the mean. The mean here is to be found not in studying wishes which a certain class of mind is fond of attributing, without real justification, to the working-class patient, but in directing every effort to the remedy of his condition: an end only to be gained, as we have seen, by the use of means already costly and complex enough.

